# Crystal structure and Hirshfeld surface analysis of the layered hybrid metal halide poly[bis­(2-iodoethyl­ammonium) [di-μ-iodido-di­iodido­germanate(II)]]

**DOI:** 10.1107/S2056989024011800

**Published:** 2025-01-01

**Authors:** Olesia I. Kucheriv, Mircea-Odin Apostu, Olena Prysiazhna, Vadim A. Potaskalov, Sergey O. Malinkin

**Affiliations:** a Department of Chemistry, Taras Shevchenko National University of Kyiv, Volodymyrska St. 64, Kyiv 01601, Ukraine; b Department of Chemistry, Faculty of Chemistry, Al. I. Cuza University of Iasi, Carol I Blvd. 11, Iasi 700506, Romania; c Bakul Institute for Superhard Materials, National Academy of Sciences of Ukraine, Avtozavodskaya St. 2, Kyiv 04074, Ukraine; d Department of Chemistry, Kyiv National University of Construction and Architecture, Povitroflotsky Ave. 31, Kyiv 03680, Ukraine; e Department of General and Inorganic Chemistry, National Technical University of Ukraine "Igor Sikorsky Kyiv Polytechnic Institute", Beresteiskyi Pr. 37, 03056 Kyiv, Ukraine; Universidad de la República, Uruguay

**Keywords:** crystal structure, hybrid perovskite, germanium(II), organic cation

## Abstract

The title compound is a germanium-based hybrid metal halide that represents a less-toxic alternative to more popular lead-based analogues in optoelectronic applications. {(2-IC_2_H_4_NH_3_)_2_[GeI_4_]}_*n*_ is composed of infinite inorganic layers that are formed by [GeI_6_]^4−^ octa­hedra connected in a corner-sharing manner with four equatorial I atoms. The organic (2-IC_2_H_4_NH_3_)^+^ cations inter­leave the inorganic layers. There are two types of 2-iodo­ethyl­ammonium cations, with synclinal and anti­periplanar conformations. The organic cations inter­act with the inorganic layers through hydrogen bonds and I⋯I contacts. The crystal under investigation was twinned by a 180° rotation around [100].

## Abstract 2

The crystal structure of the title compound contains infinite inorganic two-dimensional layers formed by [GeI_6_]^4−^ octa­hedra. These layers are inter­leaved with the organic 2-iodo­ethyl­ammonium cations.

## 1. Chemical context

The number of functional materials with exceptional physical properties has been significantly expanded by numerous hybrid organic–inorganic halide perovskites in recent decades. These materials frequently display semiconducting properties that allow their application in various photovoltaic and optoelectronic devices (Zhao & Zhu, 2016 ▸). Hybrid perovskites have been shown to form effective active layers in solar cells (Huang *et al.*, 2023 ▸), light-emitting diodes (Ngai *et al.*, 2023 ▸), photodetectors (Moeini *et al.*, 2022 ▸) and thermolectronic cells (Ngai *et al.*, 2023 ▸).

Hybrid halide perovskites are compounds of the *ABX*_3_ type (where *A* is an organic cation, *B* is a metal cation and *X* is a halide anion), which contain fundamental *BX*_6_ octa­hedra that are organized in all-corner-sharing 3D network (Akkerman & Manna, 2020 ▸; Breternitz & Schorr, 2018 ▸). Upon development of the field, numerous analogues containing *BX*_6_ octa­hedra, which are connected in various manners (corner-sharing, edge-sharing and even face-sharing), and organic cations have been developed. Such hybrid metal–halide compounds can form structures in which the inorganic octa­hedra are connected in very different manners, forming infinite layers (frequently called layered perovskites in the case of corner-to-corner connection; McNulty & Lightfoot, 2021 ▸), polymeric chains, discrete moieties and more complicated structures that include double-chains and double-layers. Hybrid metal halides of different structural motifs tend to display distinctive physical properties. For example, 3D perovskites are known to have the narrowest bandgaps and therefore are the most promising for photovoltaic and optoelectronic applications (Younis *et al.*, 2021 ▸). Layered metal halides have bandgaps which are usually *ca*. 1 eV higher than for 3D perovskites with corresponding halogens, but they display enhanced stability (Wong & Yang, 2021 ▸). Hybrid metal halides that form inorganic polymeric chains are the most efficient white-light emitters (Yuan *et al.*, 2017 ▸), while hybrid metal halides containing discrete inorganic moieties are known to be very efficient emitters with high quantum yields (Zhang *et al.*, 2021 ▸).

Importantly, the most applied and studied for today are hybrid perovskites and other metal halides that are formed with lead. However, the significant toxicity of this metal induces efforts towards research into lead-free analogues. Most frequently, lead is substituted with tin or germanium due to the chemical similarity of these elements, which allows retention of the physical properties of the target materials while reducing their toxicity.

Herein we describe the crystal structure of a new layered hybrid metal halide – (2-IC_2_H_4_NH_3_)_2_[GeI_4_]. The introduction of a 2-iodo­ethyl­ammonium cation, which is able to create I⋯I contacts, ensures additional structural stability of the framework.
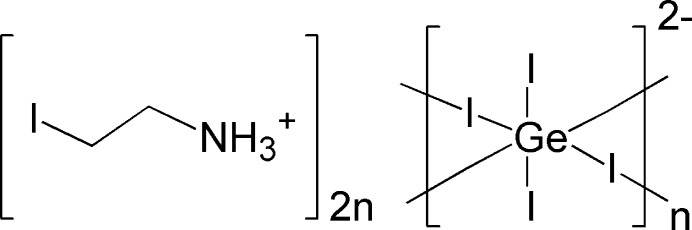


## 2. Structural commentary

**Figure 1:**
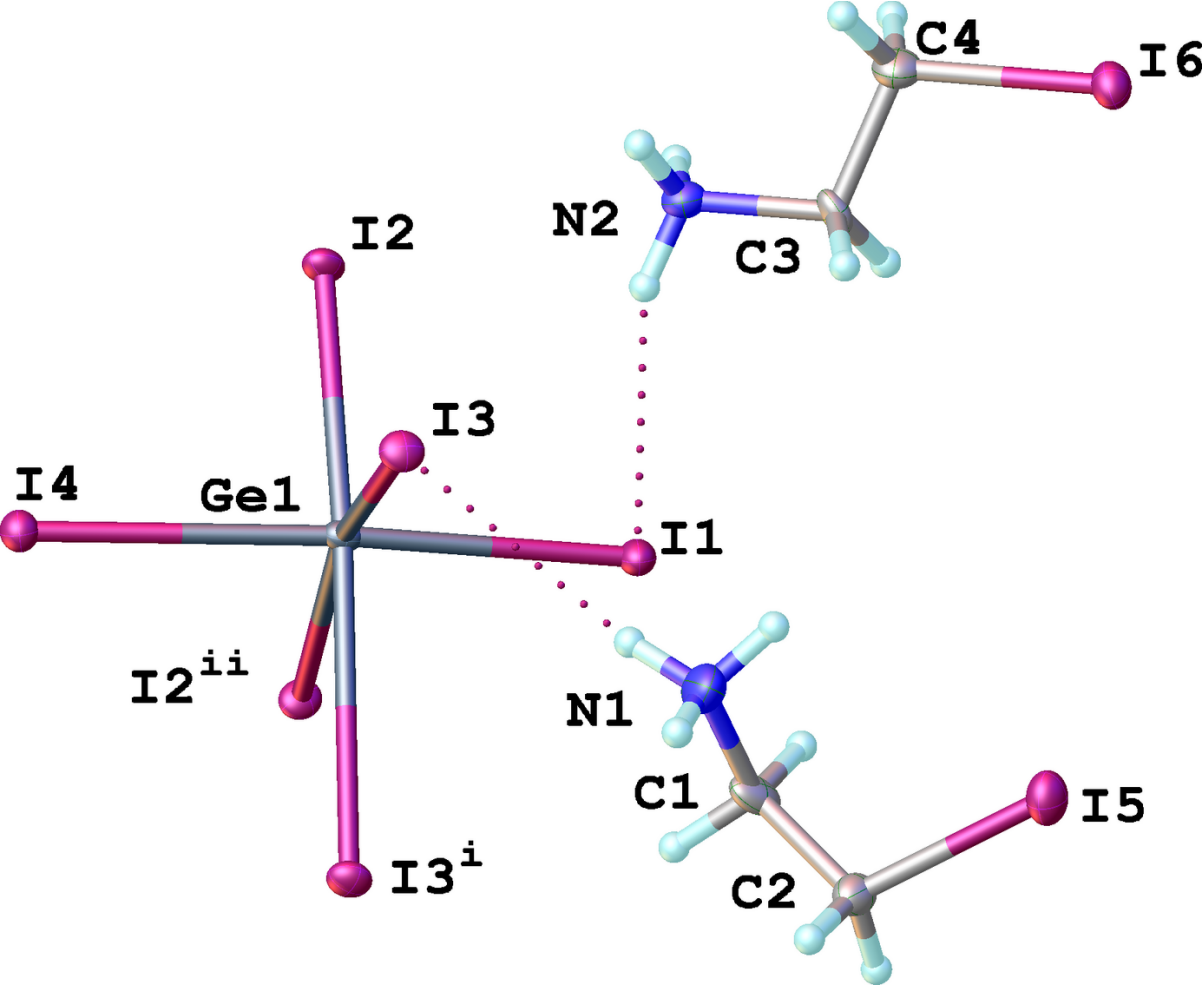
Expanded asymmetric unit of the title compound showing the atom-labeling scheme. Hydrogen bonds between organic and inorganic parts are shown as dotted lines. Displacement ellipsoids are drawn at the 50% probability level. [Symmetry codes: (i) 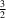 − *x*, 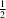 + *y*, 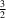 − *z*; (ii) 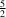 − *x*, 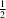 + *y*, 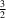 − *z*.]

The title compound (2-IC_2_H_4_NH_3_)_2_[GeI_4_] crystallizes in the centrosymmetric monoclinic *P*2_1_/*n* space group. In its crystal structure, the Ge^2+^ cation has a significantly distorted octa­hedral coordination environment provided by six iodide anions (Fig. 1 ▸). The Ge—I bond lengths range from 2.7732 (12) to 3.3646 (11) Å, resulting in an octa­hedral distortion, which can be qu­anti­fied by the quadratic elongation parameter: <λ_oct_> = Σ(l_i_/l_0_)^2^/6 = 1.0065, where *l_i_* is the individual Ge—I bond length and *l* is average Ge—I bond length (Robinson *et al.*, 1971 ▸). The *cis*-I—Ge—I angles are in the range of 75.23 (3)–95.46 (4)°, leading to a significant bond angle variance: σ_θ_^2^ = Σ(θ_i_ − 90°)^2^/11 =34.047, where θ_i_ are the twelve individual *cis*-I—Ge—I angles in the coordination octa­hedron (Robinson *et al.*, 1971 ▸). Such large distortion parameters are quite common for germanium halides. For example, in a series of layered (ClMBA)_2_GeI_4_ (ClMBA = 4-chloro-methyl­benzyl­amine) with either enanti­opure or racemic cations, bond-angle variances were in the 34.52–55.16° range, while the quadratic elongation parameter was almost the same for these materials (1.029; Coccia *et al.*, 2024 ▸). In comparison with lead, germanium has a more active lone pair, which results in significantly larger distortions.

The negative charge of the inorganic coordination octa­hedra in this structure is compensated for by the 2-iodo­ethyl­ammonium cations. There are two types of crystallographically independent cations in the structure: one of them adopts a synclinal conformation, while the other one is anti­periplanar. Torsion angles in these cations are −65.4 (11)° and 176.7 (7)°, respectively. The I—C, C—C and C—N bond lengths in the organic cations fall within the expected ranges (Allen *et al.*, 1987 ▸).

**Figure 2:**
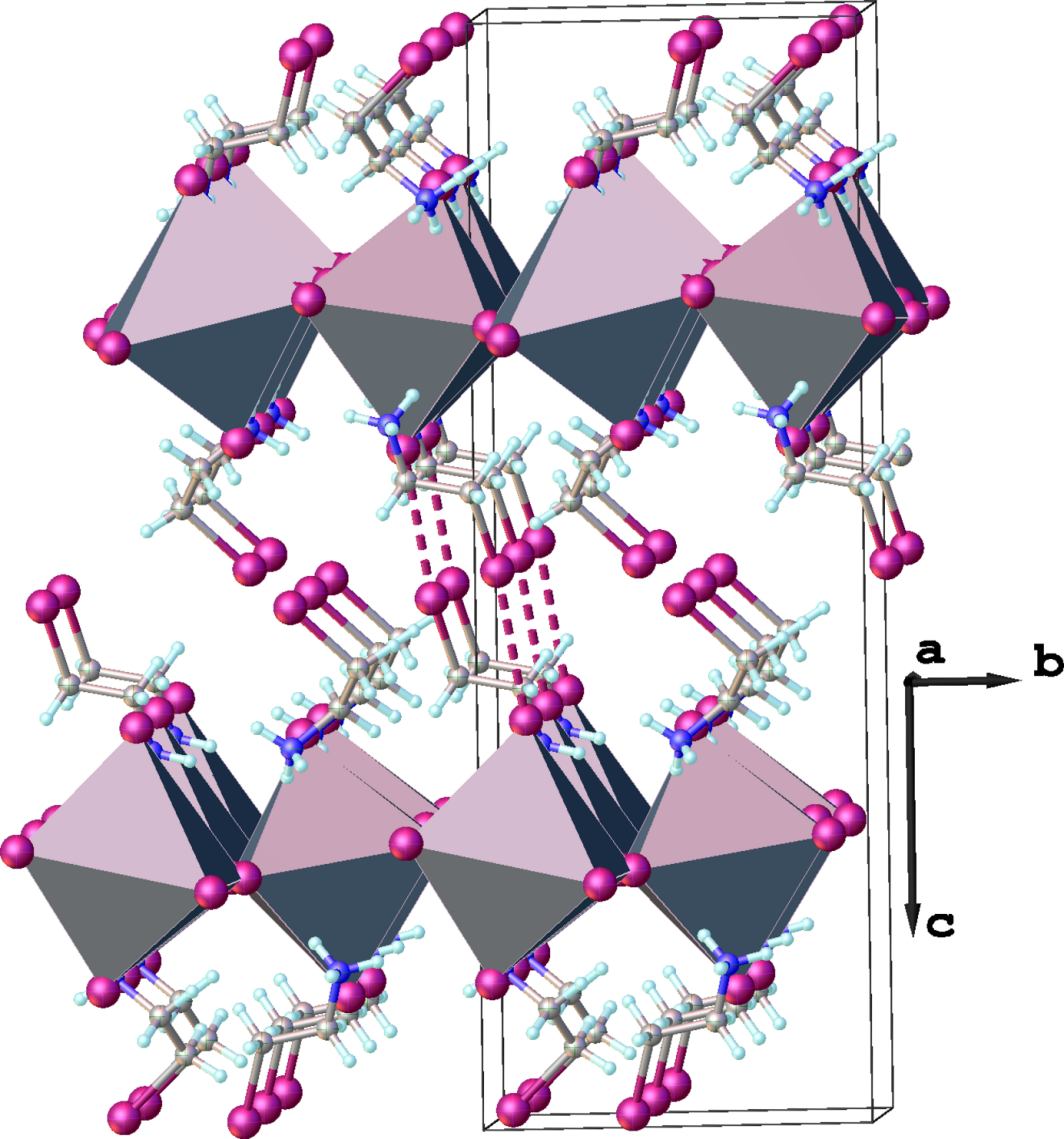
Fragment of the title compound showing the propagation of infinite two-dimensional inorganic layers along the *ab* plane. I⋯I contacts between the organic cations and the inorganic layers are shown as pink dashed lines.

The inorganic [GeI_6_]^4−^ octa­hedra connect with each other in the corner-sharing mode, creating infinite 2D layers that propagate in the *ab* plane (Fig. 2 ▸). In this type of architecture, the four equatorial iodide anions are bridging (I_b_) while two axial iodide anions are terminal (I_t_). The coordination octa­hedra undergo out-of-plane tilting with respect to the *ab* plane with the I—Ge—I angles being 161.15 (3)°, thus the layers are slightly corrugated. The organic cations are located between the inorganic layers.

## 3. Supra­molecular features

**Figure 3:**
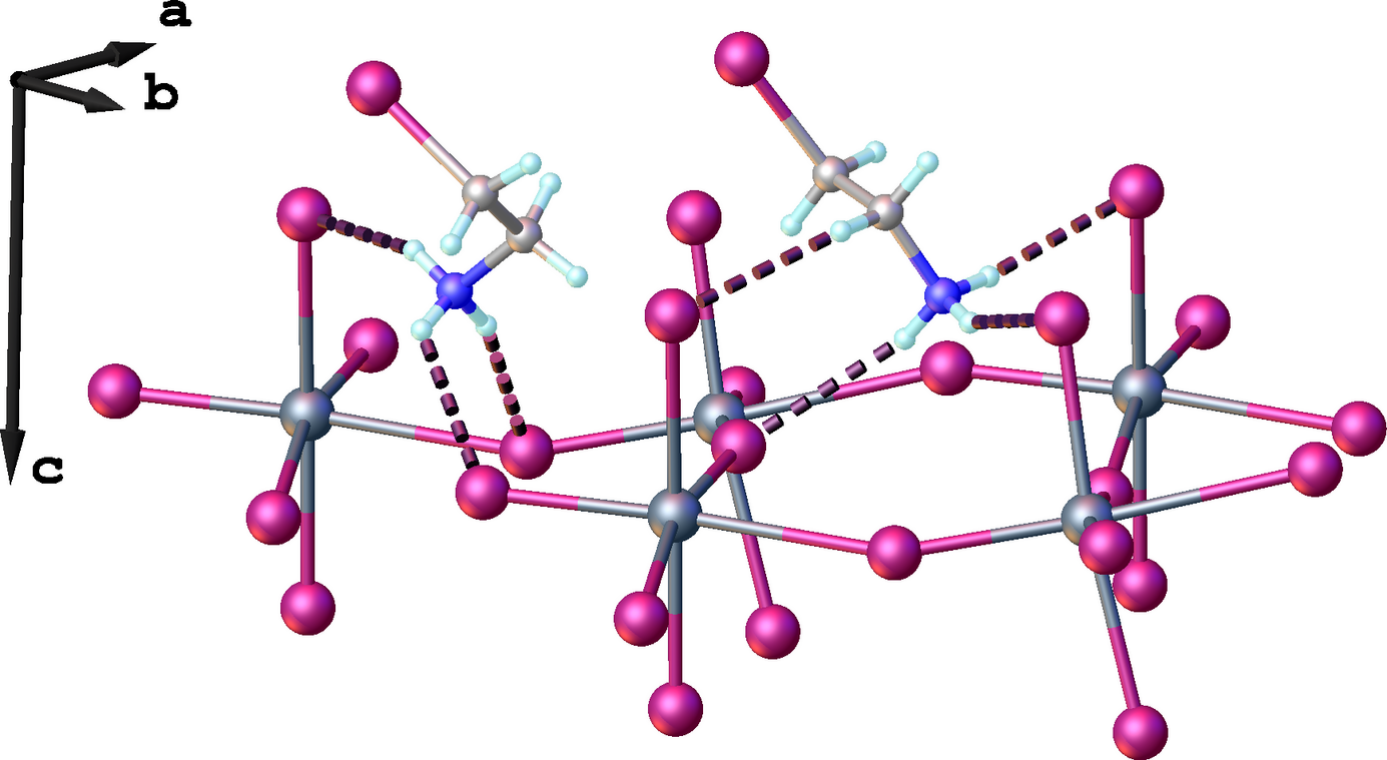
Detail of the hydrogen bonds created between the 2-iodo­ethyl­ammonium cations and the inorganic layer.

**Figure 4:**
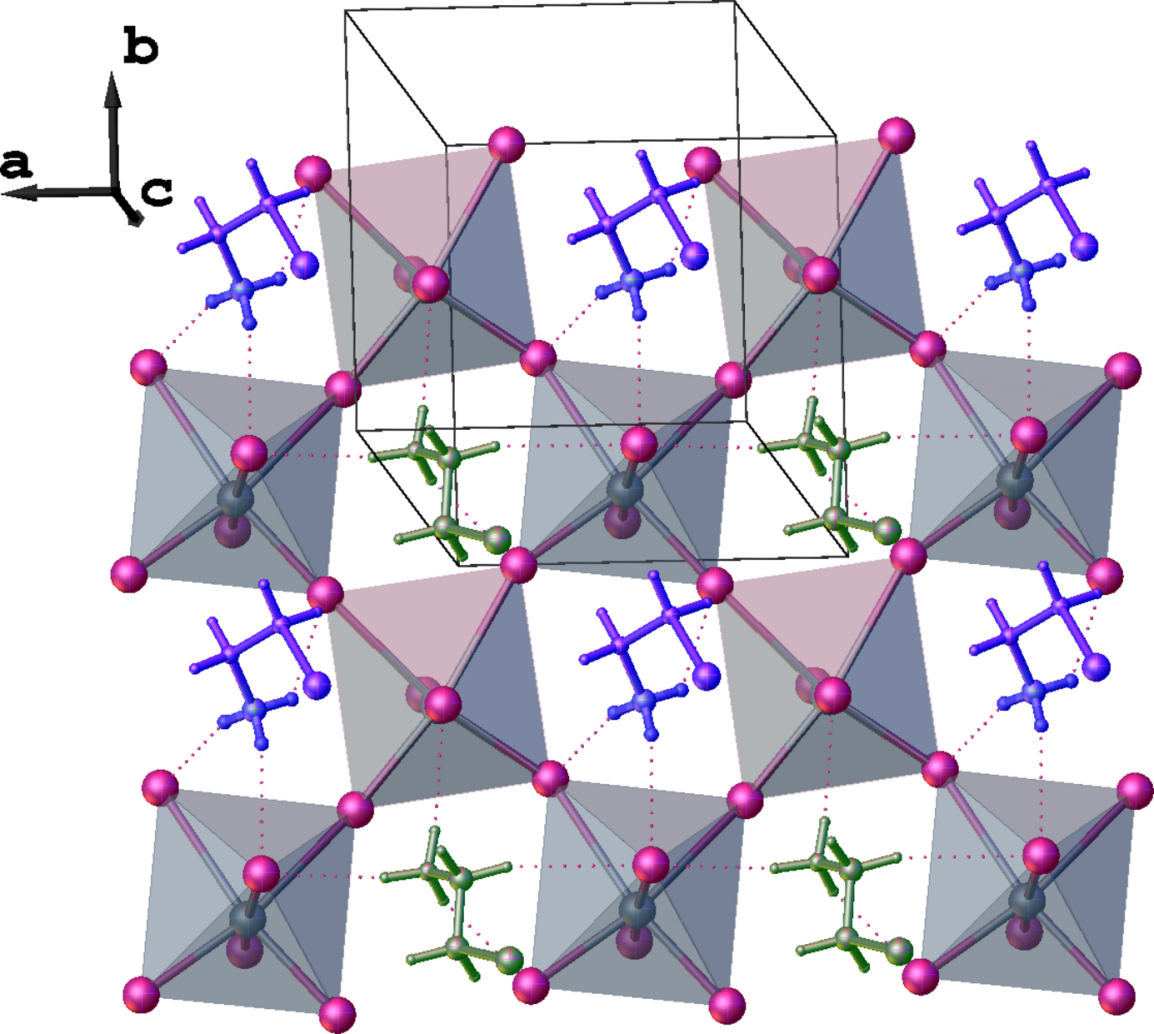
Alternating organic cations in synclinal (blue) and anti­periplanar (green) conformations within a layer.

There is an extensive network of hydrogen bonds in the title structure. The 2-iodo­ethyl­ammonium cation in a synclinal conformation forms three hydrogen bonds of the N—H⋯I type with inorganic layers: two of these hydrogen bonds involve bridging iodine atoms, while the remaining one is with a terminal iodine (Fig. 3 ▸). Detailed information about the hydrogen-bonding geometry is given in Table 1 ▸. The organic cations with an anti­periplanar conformation make four hydrogen bonds with the inorganic layers: three of them are also of the N—H⋯I type (one with a bridging iodine atom and two with terminal ones) while the fourth is of the C—H⋯I type (Fig. 2 ▸, Table 1 ▸). The cations with synclinal and anti­periplanar conformations are arranged in an alternating manner (Fig. 4 ▸). In addition, the iodine atoms of the anti­periplanar cations make I⋯I contacts with the terminal iodine atoms of neighboring inorganic layers, thus creating an infinite 3D supra­molecular framework (Fig. 2 ▸). The presence of such I⋯I contacts has previously been shown to have a significant impact on the physical properties of hybrid metal halides. For example, the presence of a very similar organic cation, 4-iodo­buthyl­ammonium, in the layered metal halide [I-(CH_2_)_4_-NH_3_]_2_PbI_4_ was found to suppress phase transitions in this material in contrast to the very similar [H-(CH_2_)_4_-NH_3_]_2_PbI_4_. Phase-transition suppression is associated with the occurrence of I⋯I inter­actions between organic cations and the inorganic layers, which restrict the movement of organic cations in space (Chakraborty *et al.*, 2022 ▸).

Inter­estingly, in contrast to the anti­periplanar conformation, the synclinal conformation of an organic cation does not permit the formation of a close I⋯I contact with the inorganic layers due to steric hindrance. Such an effect has previously been observed for a series of (ICH_2_CH_2_NH_3_)_2_(CH_3_NH_3_)_*n*-1_Pb_*n*_I_3*n*+1_ layered perovskites in which the organic cations in different conformations were shown to impact the structural symmetry and electronic band structure of the layered metal halides (Xue *et al.*, 2023 ▸).

## 4. Hirshfeld surface analysis

**Figure 5:**
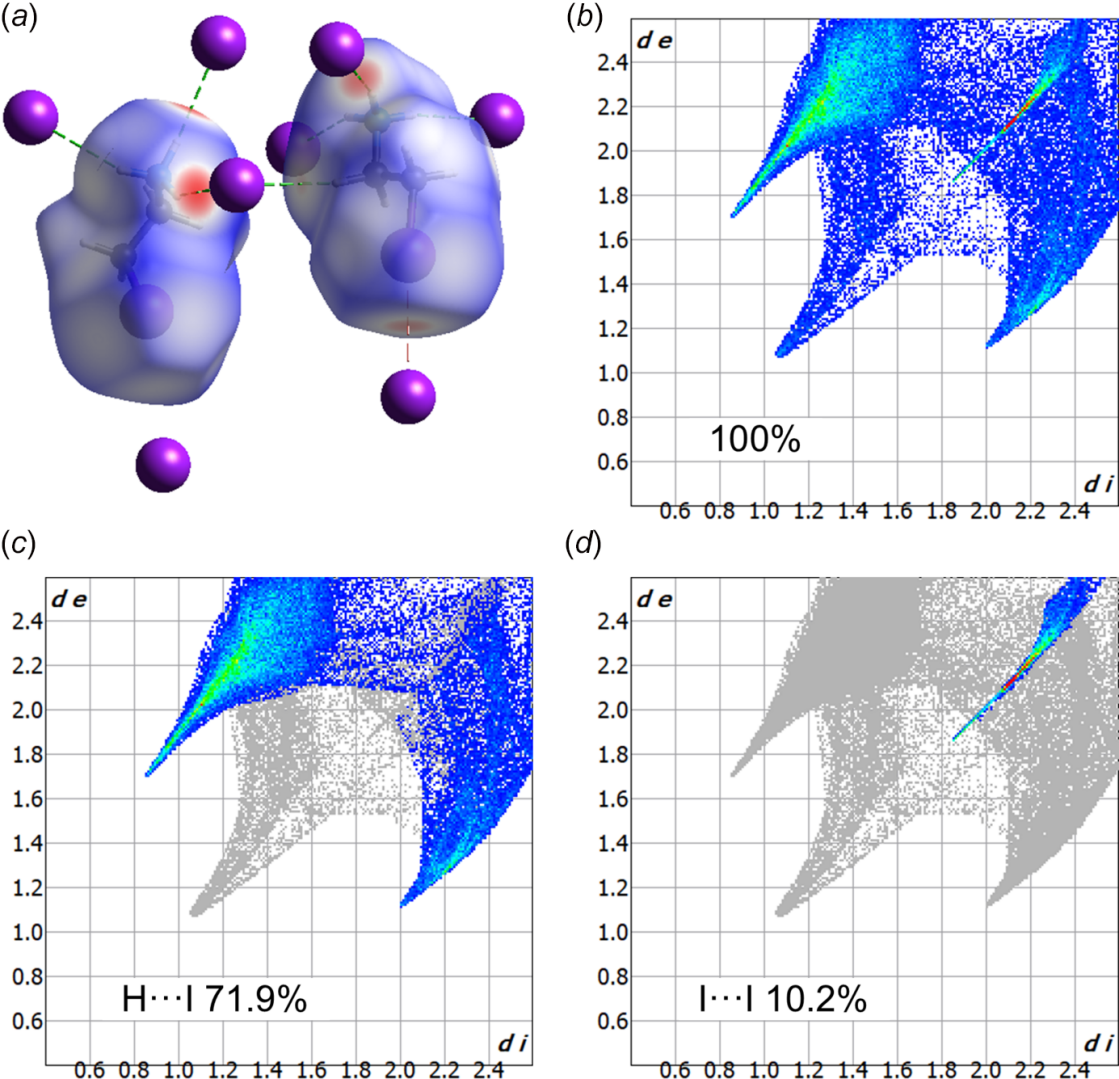
(*a*) Hirshfeld surface of the title compound plotted over *d*_norm_, showing the strongest H⋯I inter­actions in red; (*b*)–(*d*) the two-dimensional fingerprint plots for the title compound.

Hirshfeld surface analysis was performed in order to get a deeper insight into the weak inter­actions found in the structure (Hirshfeld, 1977 ▸). The Hirshfeld surface was plotted with a standard resolution of *d*_norm_ over a fixed color scale that uses white to depict contacts whose distances are close to the sum of the van der Waals radii, while shorter distances are shown in red and longer in blue. The obtained plot demonstrates the presence of several strong H⋯I contacts, shown in red in Fig. 5 ▸*a*. The above-mentioned I⋯I contacts between organic cations in an anti­periplanar conformation with the iodine atoms of the inorganic layers can be observed in pale red. The associated fingerprint plots have also been calculated (Fig. 5 ▸*b*–*d*). The plots demonstrate that H⋯I contacts are most common and constitute 71.9% of all inter­actions present in the structure (Fig. 5 ▸*c*). In addition, an important contribution is provided by I⋯I inter­actions (10.2%), as shown in Fig. 5 ▸*d*.

The prevalence of H⋯I inter­actions underscores the critical role of hydrogen bonding in stabilizing the structure, while the I⋯I contacts contribute to the three-dimensional supra­molecular framework. The remaining H⋯H contacts are statistically frequently observed in the structure due to the terminal positions of the H atoms; they are, however, chemically irrelevant.

It should be mentioned that the color map of the Hirshfeld surface displays the strength of contacts (the strongest contacts are shown in red) while the fingerprint plots indicate how frequently the corresponding contacts are observed in the structure. The Hirshfeld surface analysis was undertaken and the fingerprint plots were generated using *Crystal Explorer 21.45* software (Spackman *et al.*, 2021 ▸).

## 5. Database survey

A survey of the the Cambridge Structural Database (CSD version 5.45, update of September 2024; Groom *et al.*, 2016 ▸) showed that the title compound has never been published before. The search revealed 13 previously known structures of hybrid metal halides containing the 2-iodo­ethyl­ammonium cation. Two of these structures are (2-IC_2_H_4_NH_3_)SnI_4_ (Song *et al.*, 2022 ▸) and the mixed-cation [Br-(CH_2_)_2_-NH_3_]_2-*x*_[I-(CH_2_)_2_-NH_3_]_*x*_PbBr_*x*_I_4-*x*_ (Sourisseau *et al.*, 2007*b* ▸), which are isostructural to the title compound. There are four reported structures of (2-IC_2_H_4_NH_3_)PbI_4_, which also form inorganic layers; however in contrast to the title compound, in this lead-based analogue all of the organic cations exhibit an anti­periplanar conformation (Sourisseau *et al.*, 2007*a* ▸,*b* ▸; Lemmerer & Billing, 2010 ▸; Skorokhod *et al.*, 2023 ▸). Seven of the found structures contain a second organic cation, methyl­ammonium (MA), and can be described by the general formula (2-IC_2_H_4_NH_3_)_2_(MA)_*n*-1_Pb_*n*_I_3*n*+1_ (*n* = 2–4). In these compounds there are from two to four consecutive inorganic layers, which are connected into multilayered structures. The methyl­ammonium cations occupy positions in cubocta­hedral voids within the multilayers, while the 2-iodo­ethyl­ammonium cations separate the multilayers. When the number of layers is odd (*n* = 3), the 2-iodoethyl­ammonium cation adopts an anti­periplanar conformation, while in the case of an even number of layers (*n* = 2, 4) this cation is in a synclinal conformation (Xue *et al.*, 2023 ▸; Skorokhod *et al.*, 2023 ▸).

## 6. Synthesis and crystallization

The title compound was obtained as a sub-product upon synthesis of (aziridinium)GeI_3_; the SXRD (single-crystal X-ray diffraction) experiment established the formation of (2-IC_2_H_4_NH_3_)GeI_4_ instead of the target perovskite. 63 mg of GeO_2_ were mixed with 0.9 ml of an aqueous HI solution (57% *w*/*w*) and 0.6 ml of H_3_PO_2_ (50% *w*/*w*). The obtained mixture was heated to 393 K in an oil bath and was kept at this temperature for 20 min upon constant mixing. The obtained transparent orange solution was allowed to cool to room temperature. 10 mg of aziridine were dissolved in 0.1 ml of water and added dropwise to 0.1 ml of the prior solution. The orange crystals that formed within 1 h were collected immediately and kept in Paratone© oil prior to measurements. SXRD measurements were performed at 100 K.

## 7. Refinement

Crystal data, data collection and structure refinement details are summarized in Table 2 ▸. H atoms were placed at calculated positions and refined isotropically with *U*_iso_(H) = 1.2*U*_eq_(C) or *U*_iso_(H) = 1.2*U*_eq_(N). H atoms of secondary CH_2_ groups were refined as riding, while H atoms of NH_3_^+^ groups were refined as rotating. The crystal under investigation was twinned by a 180° rotation around [100] and the intensity data processed into a HKLF5-type file; the twin components refined to a ratio of 0.8495:0.1504.

## Supplementary Material

Crystal structure: contains datablock(s) I. DOI: 10.1107/S2056989024011800/oo2009sup1.cif

Structure factors: contains datablock(s) I. DOI: 10.1107/S2056989024011800/oo2009Isup2.hkl

CCDC reference: 2407606

Additional supporting information:  crystallographic information; 3D view; checkCIF report

## Figures and Tables

**Table 1:** Hydrogen-bond geometry (Å, °)

*D*—H⋯*A*	*D*—H	H⋯*A*	*D*⋯*A*	*D*—H⋯*A*
N1—H1*A*⋯I4^i^	0.91	2.74	3.544 (11)	147
N1—H1*B*⋯I2^ii^	0.91	3.11	3.782 (11)	133
N1—H1*C*⋯I3	0.91	2.86	3.735 (10)	162
N2—H2*A*⋯I4^iii^	0.91	2.70	3.588 (9)	166
N2—H2*B*⋯I3^i^	0.91	2.87	3.744 (9)	160
N2—H2*C*⋯I1	0.91	2.65	3.496 (9)	154
C4—H4*A*⋯I1^iv^	0.99	3.13	3.790 (10)	125

Symmetry codes: (i) 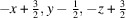; (ii) 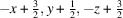; (iii) 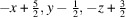; (iv) 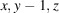.

**Table 2:** Experimental details

Crystal data	
Chemical formula	(C_2_H_7_IN)_2_[GeI_4_]
*M*_r_	924.16
Crystal system, space group	Monoclinic, *P*2_1_/*n*
Temperature (K)	100
*a*, *b*, *c* (Å)	8.1431 (2), 8.8362 (2), 25.1787 (7)
β (°)	97.264 (2)
*V* (Å^3^)	1797.16 (8)
*Z*	4
Radiation type	Mo *K*α
μ (mm^−1^)	11.99
Crystal size (mm)	0.14 × 0.08 × 0.01

Data collection	
Diffractometer	XtaLAB Synergy, Dualflex, HyPix
Absorption correction	Analytical (*CrysAlis PRO*; Rigaku OD, 2023 ▸)
*T*_min_, *T*_max_	0.399, 0.852
No. of measured, independent and observed [*I* > 2σ(*I*)] reflections	5413, 5413, 4967
*R*_int_	0.030
(sin θ/λ)_max_ (Å^−1^)	0.708

Refinement	
*R*[*F*^2^ > 2σ(*F*^2^)], *wR*(*F*^2^), *S*	0.035, 0.092, 1.19
No. of reflections	5413
No. of parameters	121
H-atom treatment	H-atom parameters constrained
Δρ_max_, Δρ_min_ (e Å^−3^)	1.21, −1.27

Computer programs: *CrysAlis PRO* (Rigaku OD, 2023 ▸), *SHELXS* (Sheldrick, 2008 ▸), *SHELXL2018/3* (Sheldrick, 2015 ▸) and *OLEX2* (Dolomanov *et al.*, 2009 ▸).

## supplementary crystallographic information

### Poly[bis(2-iodoethylammonium) [di-µ-iodido-diiodidogermanate(II)]] Crystal data

**Table:**

(C_2_H_7_IN)_2_[GeI_4_]	*F*(000) = 1608
*M**_r_* = 924.16	*D*_x_ = 3.416 Mg m^−^^3^
Monoclinic, *P*2_1_/*n*	Mo *K*α radiation, λ = 0.71073 Å
*a* = 8.1431 (2) Å	Cell parameters from 10013 reflections
*b* = 8.8362 (2) Å	θ = 3.3–30.5°
*c* = 25.1787 (7) Å	µ = 11.99 mm^−^^1^
β = 97.264 (2)°	*T* = 100 K
*V* = 1797.16 (8) Å^3^	Plate, clear intense orange
*Z* = 4	0.14 × 0.08 × 0.01 mm

### Poly[bis(2-iodoethylammonium) [di-µ-iodido-diiodidogermanate(II)]] Data collection

**Table:**

XtaLAB Synergy, Dualflex, HyPix diffractometer	4967 reflections with *I* > 2σ(*I*)
Detector resolution: 10.0000 pixels mm^-1^	*R*_int_ = 0.030
ω scans	θ_max_ = 30.2°, θ_min_ = 2.4°
Absorption correction: analytical (CrysAlisPro; Rigaku OD, 2023)	*h* = −10→11
*T*_min_ = 0.399, *T*_max_ = 0.852	*k* = −11→11
5413 measured reflections	*l* = −31→31
5413 independent reflections	

### Poly[bis(2-iodoethylammonium) [di-µ-iodido-diiodidogermanate(II)]] Refinement

**Table:**

Refinement on *F*^2^	0 restraints
Least-squares matrix: full	Hydrogen site location: inferred from neighbouring sites
*R*[*F*^2^ > 2σ(*F*^2^)] = 0.035	H-atom parameters constrained
*wR*(*F*^2^) = 0.092	*w* = 1/[σ^2^(*F*_o_^2^) + (0.0215*P*)^2^ + 42.6836*P*] where *P* = (*F*_o_^2^ + 2*F*_c_^2^)/3
*S* = 1.19	(Δ/σ)_max_ = 0.001
5413 reflections	Δρ_max_ = 1.21 e Å^−^^3^
121 parameters	Δρ_min_ = −1.27 e Å^−^^3^

### Poly[bis(2-iodoethylammonium) [di-µ-iodido-diiodidogermanate(II)]] Special details

**Table:**

Geometry. All esds (except the esd in the dihedral angle between two l.s. planes) are estimated using the full covariance matrix. The cell esds are taken into account individually in the estimation of esds in distances, angles and torsion angles; correlations between esds in cell parameters are only used when they are defined by crystal symmetry. An approximate (isotropic) treatment of cell esds is used for estimating esds involving l.s. planes.
Refinement. Refined as a 2-component twin.

### Poly[bis(2-iodoethylammonium) [di-µ-iodido-diiodidogermanate(II)]] Fractional atomic coordinates and isotropic or equivalent isotropic displacement parameters (Å^2^)

**Table:**

	*x*	*y*	*z*	*U*_iso_*/*U*_eq_	
I2	1.22418 (8)	0.35127 (7)	0.76979 (3)	0.01606 (14)	
I3	0.72110 (8)	0.41154 (7)	0.76396 (3)	0.01657 (14)	
I1	0.95890 (8)	0.55334 (7)	0.63911 (3)	0.01699 (14)	
I4	1.06654 (8)	0.65550 (7)	0.87689 (3)	0.01740 (14)	
I6	0.77665 (9)	−0.09924 (8)	0.51564 (3)	0.02055 (15)	
I5	0.25037 (9)	0.56475 (9)	0.51448 (3)	0.02426 (16)	
Ge1	0.99725 (12)	0.58843 (11)	0.75456 (4)	0.0128 (2)	
N1	0.4449 (15)	0.5521 (12)	0.6444 (4)	0.035 (3)	
H1A	0.423703	0.465891	0.624939	0.042*	
H1B	0.352259	0.580595	0.658589	0.042*	
H1C	0.528452	0.534821	0.671279	0.042*	
C3	0.9157 (12)	0.1243 (11)	0.6000 (4)	0.016 (2)	
H3A	0.957632	0.183196	0.571113	0.019*	
H3B	0.798169	0.151794	0.600885	0.019*	
N2	1.0135 (11)	0.1621 (10)	0.6523 (4)	0.0201 (19)	
H2A	1.123115	0.148652	0.649940	0.024*	
H2B	0.982275	0.100583	0.678197	0.024*	
H2C	0.994867	0.260296	0.660675	0.024*	
C2	0.3521 (13)	0.7312 (12)	0.5719 (5)	0.022 (2)	
H2D	0.388261	0.820189	0.552453	0.026*	
H2E	0.264226	0.765182	0.592961	0.026*	
C4	0.9289 (15)	−0.0421 (12)	0.5888 (5)	0.024 (2)	
H4A	0.893086	−0.100939	0.618776	0.028*	
H4B	1.045603	−0.068378	0.585890	0.028*	
C1	0.4942 (13)	0.6745 (12)	0.6091 (5)	0.024 (2)	
H1D	0.543005	0.759248	0.631442	0.029*	
H1E	0.580109	0.635510	0.588073	0.029*	

### Poly[bis(2-iodoethylammonium) [di-µ-iodido-diiodidogermanate(II)]] Atomic displacement parameters (Å^2^)

**Table:**

	*U*^11^	*U*^22^	*U*^33^	*U*^12^	*U*^13^	*U*^23^
I2	0.0138 (3)	0.0141 (3)	0.0197 (3)	0.0044 (2)	0.0000 (2)	−0.0016 (2)
I3	0.0131 (3)	0.0153 (3)	0.0213 (3)	−0.0038 (2)	0.0023 (2)	0.0007 (2)
I1	0.0233 (3)	0.0132 (3)	0.0140 (3)	0.0010 (2)	0.0006 (3)	0.0001 (2)
I4	0.0192 (3)	0.0172 (3)	0.0155 (3)	−0.0001 (3)	0.0012 (3)	−0.0008 (2)
I6	0.0244 (3)	0.0201 (3)	0.0170 (3)	−0.0036 (3)	0.0019 (3)	−0.0022 (3)
I5	0.0223 (3)	0.0297 (4)	0.0197 (4)	−0.0009 (3)	−0.0015 (3)	−0.0035 (3)
Ge1	0.0112 (4)	0.0101 (4)	0.0172 (5)	0.0000 (4)	0.0016 (4)	0.0002 (4)
N1	0.056 (7)	0.029 (5)	0.019 (5)	0.011 (5)	−0.003 (5)	0.000 (4)
C3	0.016 (4)	0.017 (5)	0.016 (5)	−0.001 (4)	0.004 (4)	−0.008 (4)
N2	0.025 (5)	0.015 (4)	0.020 (5)	0.002 (3)	0.001 (4)	0.002 (3)
C2	0.024 (5)	0.017 (5)	0.022 (6)	−0.002 (4)	−0.004 (4)	0.000 (4)
C4	0.030 (6)	0.015 (5)	0.025 (6)	−0.001 (4)	0.001 (5)	0.002 (4)
C1	0.021 (5)	0.014 (5)	0.035 (7)	−0.001 (4)	−0.006 (5)	−0.008 (5)

### Poly[bis(2-iodoethylammonium) [di-µ-iodido-diiodidogermanate(II)]] Geometric parameters (Å, º)

**Table:**

I2—Ge1	2.7881 (11)	C3—N2	1.487 (13)
I3—Ge1	2.7732 (12)	C3—C4	1.503 (14)
I1—Ge1	2.9006 (12)	N2—H2A	0.9100
I4—Ge1	3.1172 (12)	N2—H2B	0.9100
I6—C4	2.147 (11)	N2—H2C	0.9100
I5—C2	2.153 (10)	C2—H2D	0.9900
N1—H1A	0.9100	C2—H2E	0.9900
N1—H1B	0.9100	C2—C1	1.481 (14)
N1—H1C	0.9100	C4—H4A	0.9900
N1—C1	1.488 (16)	C4—H4B	0.9900
C3—H3A	0.9900	C1—H1D	0.9900
C3—H3B	0.9900	C1—H1E	0.9900
			
I2—Ge1—I1	92.47 (4)	H2A—N2—H2B	109.5
I2—Ge1—I4	88.45 (3)	H2A—N2—H2C	109.5
I3—Ge1—I2	95.46 (4)	H2B—N2—H2C	109.5
I3—Ge1—I1	92.27 (3)	I5—C2—H2D	108.9
I3—Ge1—I4	94.03 (4)	I5—C2—H2E	108.9
I1—Ge1—I4	173.52 (4)	H2D—C2—H2E	107.7
H1A—N1—H1B	109.5	C1—C2—I5	113.4 (7)
H1A—N1—H1C	109.5	C1—C2—H2D	108.9
H1B—N1—H1C	109.5	C1—C2—H2E	108.9
C1—N1—H1A	109.5	I6—C4—H4A	109.6
C1—N1—H1B	109.5	I6—C4—H4B	109.6
C1—N1—H1C	109.5	C3—C4—I6	110.1 (7)
H3A—C3—H3B	108.2	C3—C4—H4A	109.6
N2—C3—H3A	109.7	C3—C4—H4B	109.6
N2—C3—H3B	109.7	H4A—C4—H4B	108.2
N2—C3—C4	110.0 (9)	N1—C1—H1D	109.2
C4—C3—H3A	109.7	N1—C1—H1E	109.2
C4—C3—H3B	109.7	C2—C1—N1	112.1 (9)
C3—N2—H2A	109.5	C2—C1—H1D	109.2
C3—N2—H2B	109.5	C2—C1—H1E	109.2
C3—N2—H2C	109.5	H1D—C1—H1E	107.9
			
I5—C2—C1—N1	−65.4 (11)	N2—C3—C4—I6	176.7 (7)

### Poly[bis(2-iodoethylammonium) [di-µ-iodido-diiodidogermanate(II)]] Hydrogen-bond geometry (Å, º)

**Table:**

*D*—H···*A*	*D*—H	H···*A*	*D*···*A*	*D*—H···*A*
N1—H1*A*···I4^i^	0.91	2.74	3.544 (11)	147
N1—H1*B*···I2^ii^	0.91	3.11	3.782 (11)	133
N1—H1*C*···I3	0.91	2.86	3.735 (10)	162
N2—H2*A*···I4^iii^	0.91	2.70	3.588 (9)	166
N2—H2*B*···I3^i^	0.91	2.87	3.744 (9)	160
N2—H2*C*···I1	0.91	2.65	3.496 (9)	154
C4—H4*A*···I1^iv^	0.99	3.13	3.790 (10)	125

Symmetry codes: (i) −*x*+3/2, *y*−1/2, −*z*+3/2; (ii) −*x*+3/2, *y*+1/2, −*z*+3/2; (iii) −*x*+5/2, *y*−1/2, −*z*+3/2; (iv) *x*, *y*−1, *z*.
